# Effects of a programme of vigorous physical activity during secondary school physical education on academic performance, fitness, cognition, mental health and the brain of adolescents (Fit to Study): study protocol for a cluster-randomised trial

**DOI:** 10.1186/s13063-019-3279-6

**Published:** 2019-04-02

**Authors:** T. M. Wassenaar, C. M. Wheatley, N. Beale, P. Salvan, A. Meaney, J. B. Possee, K. E. Atherton, J. L. Duda, H. Dawes, H. Johansen-Berg

**Affiliations:** 1 Wellcome Centre for Integrative Neuroimaging, FMRIB Centre, Nuffield Department of Clinical Neurosciences, University of Oxford, John Radcliffe Hospital, Headley Way, Oxford, OX3 9DU UK; 2 Centre for Movement, Occupational and Rehabilitation Sciences (MOReS), Oxford Brookes University, Headington Campus, Oxford, OX3 0BP UK; 3 School of Sport, Exercise and Rehabilitation Sciences, University of Birmingham, Birmingham, B15 2TT UK

**Keywords:** Cluster-randomised trial, Adolescence, Exercise, Physical activity, Fitness, Academic achievement, Cognitive functions, Mental health, MRI

## Abstract

**Background:**

Early adolescence is a period of dynamic neurobiological change. Converging lines of research suggest that regular physical activity (PA) and improved aerobic fitness have the potential to stimulate positive brain changes, improve cognitive function and boost academic attainment in this age group, but high-quality studies are needed to substantiate these findings. The primary aim of the Fit to Study trial is to investigate whether short infusions of vigorous PA (VPA) delivered during secondary school physical education (PE) can improve attainment in maths, as described in a protocol published by NatCen Social Research. The present protocol concerns the trial’s secondary outcome measures, which are variables thought to moderate or mediate the relationship between PA and attainment, including the effect of the intervention on cardiorespiratory fitness, cognitive performance, mental health and brain structure and function.

**Method:**

The Fit to Study project is a cluster-randomised controlled trial that includes Year 8 pupils (aged 12–13) from secondary state schools in South/Mid-England. Schools were randomised into an intervention condition in which PE teachers delivered an additional 10 min of VPA per PE lesson for one academic year, or a ‘PE as usual’ control condition. Intervention and control groups were stratified according to whether schools were single-sex or co-educational. Assessments take place at baseline (end of Year 7, aged 11–12) and after 12 months (Year 8). Secondary outcomes are cardiorespiratory fitness, objective PA during PE, cognitive performance and mental health. The study also includes exploratory measures of daytime sleepiness, attitudes towards daily PA and PE enjoyment. A sub-set of pupils from a sub-set of schools will also take part in a brain imaging sub-study, which is embedded in the trial.

**Discussion:**

The Fit to Study trial could advance our understanding of the complex relationships between PA and aerobic fitness, the brain, cognitive performance, mental health and academic attainment during adolescence. Further, it will add to our understanding of whether school PE is an effective setting to increase VPA and fitness, which could inform future PA interventions and education policy.

**Trial registration:**

ClinicalTrials.gov, NCT03286725. Retrospectively registered on 18 September 2017.

ClinicalTrials.gov, NCT03593863. Retrospectively registered on 19 July 2018.

**Electronic supplementary material:**

The online version of this article (10.1186/s13063-019-3279-6) contains supplementary material, which is available to authorized users.

## Background

Early adolescence is a period of dynamic neurobiological and psychological change [1] and provides a foundation for future health [2]. Understanding how health and education policies might steer the developmental course in a positive direction is therefore important [3].

Converging lines of research suggest that physical activity (PA) and aerobic exercise have the potential to stimulate positive brain changes, improve cognitive function and boost academic attainment in this age group. Neuroscience studies using animal models have indicated that, at the molecular level, aerobic exercise increases levels of growth factors responsible for synaptic plasticity, particularly in the hippocampus [4, 5]. At the cellular level, increased growth factor production is thought to promote the development of new blood vessels and neurons and their integration into existing networks of cells in this region [6]. Neuroimaging studies in adults [7], and increasingly in children [8, 9], have found support for PA and fitness-related changes in brain function and structure. For instance, hippocampal volume is larger in higher fit children and may mediate the relationship between fitness level and memory outcomes [10].

Meanwhile, intervention studies in schools have found some evidence that programmes of aerobic exercise can lead to improved cognitive performance, particularly in the domains of attention and executive functions (working memory, cognitive flexibility, and inhibitory control) [11–17]. Separately, there are associations between objectively measured PA and academic attainment among adolescents [18] and indications that school-based exercise interventions can improve educational outcomes in this age group [9, 19]. The balance of evidence among adolescents favours positive associations between aerobic exercise, fitness, cognition and attainment [20], although the variety of study designs, including different types, intensities and durations of PA, makes firm conclusions difficult to draw.

The benefits of PA for physical and mental health are well known [21, 22], yet PA levels among young adolescents are low [23] and continue to decline during early adolescence [24]. Adolescence is also a peak time for the onset of mental ill-being, including vulnerability to low self-esteem [25, 26]. There is some evidence that physical health and mental well-being and ill-being are also linked with academic attainment [27, 28], but whether these factors act as mediators in the relationship between PA and academic attainment is currently unclear.

School is a recommended setting for promoting PA among adolescents, including those from lower socio-economic backgrounds, because they spend a large proportion of their time at school and interventions can be delivered without relying on support from families [29]. There is evidence that physical education (PE) interventions to increase activity are an effective method of improving cognitive function and academic performance [11, 19]. However, less than 50% of a typical PE lesson is spent in moderate-to-vigorous PA (MVPA), the type of activities that noticeably accelerate the heart rate [30, 31]. Short bouts of high-intensity or vigorous physical activity (VPA), during which individuals work at around 80% of their maximum heart rate for between 45 s and 4 min, have been shown to deliver equivalent fitness benefits to longer, lower-intensity workouts [32, 33]. This type of vigorous activity is brief enough to be incorporated into PE lessons without disrupting curriculum delivery, and there is evidence that it can improve adolescent fitness [34, 35].

The primary aim of the trial is to investigate the impact of a 1-year VPA intervention (Fit to Study) delivered during PE on maths attainment. To date there is little school-based evidence to test whether relationships between PA and academic achievement among young adolescents are mediated or moderated by fitness, cognitive function, mental health outcomes and/or the brain. The secondary aim of this trial is therefore to investigate whether the intervention improves fitness, cognitive performance and mental health outcomes, and changes brain structure and function, compared to usual PE lessons delivered over a school year, and to further explore relationships between these measures.

### Hypotheses

The primary hypothesis for the trial is:

1. A programme of VPA, delivered during PE lessons for one academic year (10 months), will improve maths attainment at the end of the intervention in the intervention group relative to the control group.

The secondary hypotheses are:

2. The intervention will increase VPA during PE, and fitness levels, of pupils in the intervention group relative to the control group.
3. The intervention will improve cognitive performance (processing speed, memory and executive functions) in the intervention group relative to the control group.
4. The intervention will improve physical and mental health outcomes in the intervention group relative to the control group.
5. The intervention will change brain structure and function in the intervention group relative to the control group.

## Methods and analysis

### Design

**Fig. 1:**
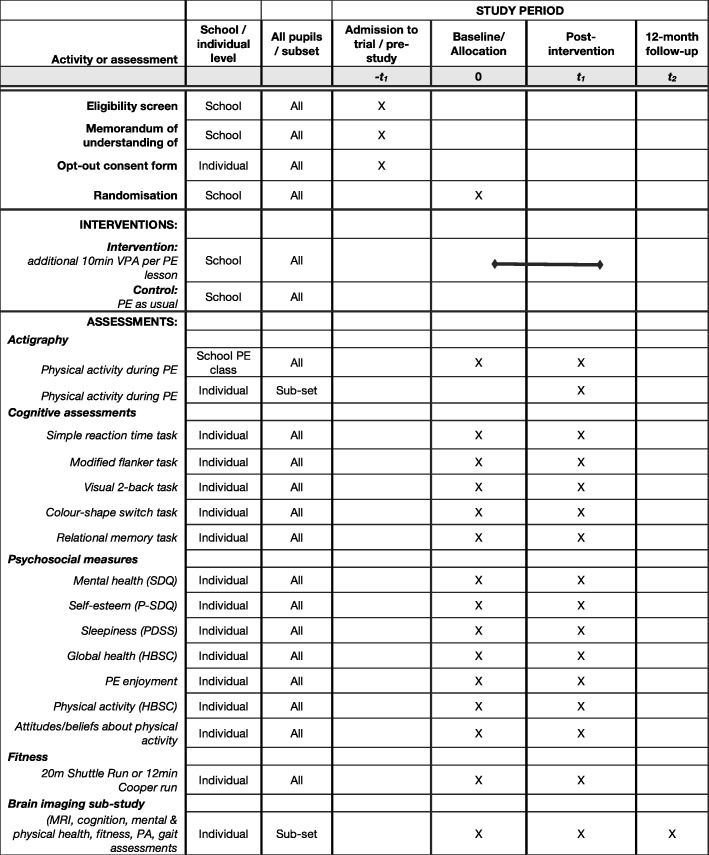
Fit to Study trial schedule of forms and procedures (adapted from SPIRIT figure). Abbreviations: *HBSC* Health Behaviour in School-aged Children, *PDSS* Paediatric Daytime Sleepiness Scale, *PE* physical education, *P-SDQ* Physical Self-Description Questionnaire, *SDQ* Strengths and Difficulties Questionnaire, *PA* physical activity, *VPA* vigorous physical activity

The study is a parallel group, superiority cluster-randomised efficacy trial of a one academic year (10-month) VPA intervention versus control. Clusters are secondary state schools in South/Mid-England; participants are Year 8 pupils within those schools. The intervention is incorporated into PE lessons to minimally disturb the curriculum and provide a scalable intervention that could be easily implemented in regular school PE lessons. Trial data are collected at baseline (end of Year 7, pupils aged 11–12) and after 12 months (end of Year 8, pupils aged 12–13). Figure 1 presents an overview of the schedule for enrolment, the intervention and the assessments, according to the Standard Protocol Items: Recommendations for Interventional Trials (SPIRIT) guidelines (Additional file 1).

A sub-set of pupils from a sub-set of schools will also take part in a brain imaging sub-study which is embedded in the trial. Data for the brain imaging sub-study are collected at baseline (end of Year 7, pupils aged 11–12), after 12 months (end of Year 8, pupils aged 12–13) and at 24 months (end of Year 9, pupils aged 13–14).

The trial has been approved by the Central University Research Ethics Committee of Oxford University (Registration No. R48879). The main trial protocol was retrospectively registered at ClinicalTrials.gov on 18 September 2017 (NCT03286725). At this time, study enrolment was complete and data acquisition was in progress. The brain imaging sub-study protocol was retrospectively registered on 19 July 2018 (NCT03593863). At this time study enrolment and data acquisition were both still in progress. The primary measure, attainment in maths, is recorded separately on the ISRCTN registry (15730512).

### Independent evaluation

The trial will be independently evaluated by NatCen Social Research. NatCen performed the randomisation and the trial’s power calculation, and will carry out the primary intention-to-treat analysis, comparing maths performance between intervention and control schools. The protocol and statistical analysis plan for the independent evaluation has been published separately [36, 37].

### Sample selection and recruitment

**Fig. 2:**
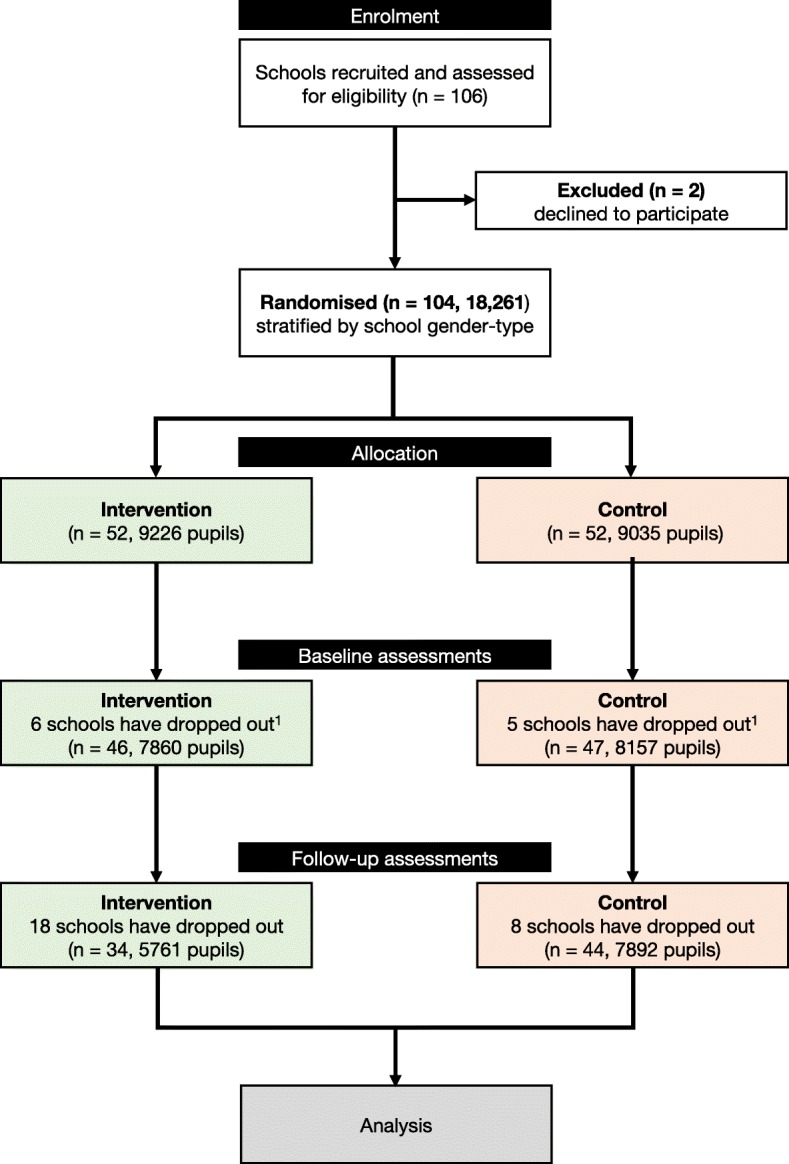
Flow diagram of schools and participants. ^1^A total of 104 schools were randomised into an intervention and a control group. Prior to baseline assessments, 11 schools dropped out. Schools were informed of their assigned group by the research team after completing baseline assessments

A total of 106 schools have been recruited and assessed for eligibility (Fig. 2). To be eligible, schools must (1) be secondary state or academy schools, but not grammar schools; (2) be mixed or single-gender; (3) have a proportion of pupils eligible for free school meals (FSM), preferably more than 15%, which is the average for England (at the time of recruitment [36]); (4) be located within a pre-defined set of local authorities, encompassing the following geographical locations: Greater London; Thames Valley; Southampton and Portsmouth; Bristol and Bath; Birmingham and Coventry; Cheltenham/Gloucester; Luton, Bedford Milton Keynes; (5) have Year 7 pupils who will move on to Year 8 at the start of the intervention; (6) deliver PE as part of their curriculum; (7) sign an agreement to send opt-out consent forms to parents/carers of Year 7 pupils and inform the research team of pupils who have opted out of data storage. Following the eligibility assessment, two schools declined to participate. Of the remaining 104 schools, all pupils in Year 8 at the start of the intervention (academic year 2017–2018) are part of the trial, given that project activities are classed as part of regular school activities. However, pupils who opted out of data storage are not required to participate in any of the trial’s assessments, unless decided otherwise by the PE teacher.

The National Foundation for Educational Research (NFER) led recruitment, supported by Oxford Brookes University. The NFER initially sent recruitment materials to 1125 schools, and, to improve response numbers, widened their search area to include another 223 schools. Recruitment presentations were given at County PE Teacher Conferences organised by the Oxfordshire Sports Partnership and the Buckinghamshire and Milton Keynes Sport and Activity Partnership. Those schools that wished to participate and met the inclusion criteria provided the names, dates of birth, genders, FSM status and unique pupil numbers (UPNs) of all Year 7 pupils. Schools were considered formally recruited upon transfer of the data to the NFER.

### Randomisation

Schools (*n* = 104) were randomised into an intervention group or control ‘PE as usual’ control group using stratified block randomisation and an allocation ratio of 1:1 (Fig. 2). Stratification was arranged by single-sex/co-educational status of schools. NatCen performed the randomisation using a random number generator in STATA (v.12) [36, 37]. To reduce the risk of bias, schools were informed of their assigned group by the research team after completing baseline assessments.

### Intervention

The intervention consisted of a one academic year (10 months: September 2017–June 2018) PA programme delivered by PE teachers during regular Year 8 PE lessons. In the summer term prior to the trial, Year 8 PE staff from all intervention schools were invited to attend a 2-h face-to-face training session delivered by a team from Oxford Brookes University who designed the intervention. Those unable to attend were asked to join an interactive 2-h online training session delivering the same content. Teachers unable to take part during the summer term attended training at the start of the autumn term. During these sessions, the intervention was described to the teachers.

The intervention requires teachers to deliver specific elements of additional VPA, over and above normal VPA, during all Year 8 PE lessons, for the whole school year. Four minutes of VPA are incorporated into the PE lesson as part of a warm-up, and three 2-min infusions per hour of PE are incorporated into the main PE class. In this study, VPA is defined as activities that raise the heart rate (HR) to 71–85% of the maximum HR (HRmax), whereas moderate PA raises it to 60–70% of HRmax. The intervention elements are designed to be easy to incorporate into PE lessons, to maximise the training effect and to minimise the risk of injury with a warm-up that progresses from light to moderate-intensity activities and includes bursts of VPA.

Teachers were asked to deliver an active 10-min warm-up at the start of each lesson, beginning with light-intensity movements (e.g. wrist rotations; leg swings) and moving through moderate-intensity activity (e.g. arm rotations; walking with high knee-raise) to incorporate two periods of VPA (e.g. vigorous arm sprints; running on the spot) totalling 4 min. Fifteen young adolescents piloted the warm-up and the infusions while wearing chest strap Polar heart rate monitors, which indicated that heart rates were in the vigorous zone for the specified time during all the intervention elements.

In addition, three 2-min infusions of VPA would be incorporated into the main PE lesson, assuming a 1-h lesson duration. If lessons were shorter or longer than 1 h, then the number of infusions was scaled up or down accordingly. Teachers were provided with three example infusions: infusion 1 consisted of fast arm rotations (the aim is to build the intensity by speeding up arm movements concluding with two maximum effort sprints for counts of ten); infusion 2 comprised squats and lunges with an emphasis on technique and a full range of motion (to work the muscles to the maximum, concluding with a maximum effort sprint on the spot for a count of ten); and infusion 3 was sprinting on the spot (this must be completed at a vigorous intensity with the two ‘on the spot’ sprint sessions lasting for counts of ten), with active recovery periods where necessary during each 2-min session. Teachers were also invited to create their own warm-up and/or infusions to provide similar levels of VPA to fit their lesson requirements. Researchers explained the rationale for the intervention and discussed implementation strategies.

Teachers were given access to video demonstrations of the warm-up and infusion material on the trial’s website. Heads of PE at intervention schools received emails reminding them to deliver the intervention in September and December 2017 and in February 2018. Researchers visited 30 intervention schools between September and December 2017 to answer questions and offer support and advice. PE staff were invited to join an online intervention discussion forum, and schools were offered the chance to have intervention lessons filmed. Researchers were also available to answer email and phone queries. Control schools were not given training and were asked to deliver PE as usual. During the intervention period, heads of PE in these schools received emails in September 2017 and February 2018 with information about testing. School participation was incentivised to minimise drop-out and ensure compliance. Both intervention and control schools receive £500 upon completion of the trial, following the academic attainment assessment.

### Intervention fidelity

All Year 8 PE teachers in intervention schools were asked to keep a written log of VPA achieved in each PE lesson. Specifically, they were asked to record whether the class completed 4 min of VPA during the warm-up and to mark how many infusions were delivered. Teachers were informed that for a teacher-created warm-up to be marked as completed, it must have lasted 10 min and progressed from light-intensity small movement activities to larger moderate-intensity activities with short bursts of vigorous-intensity activities. For a teacher-created infusion to be marked as completed, it must have lasted 2 min and included two short bursts of VPA (or equivalent, as per infusion 2, described in the ’Intervention’ section).

Further, researchers are visiting a convenience sample of intervention and control schools during 2018 to take objective measurements of PA during PE using Axivity AX3 accelerometers (see ‘Physical activity during PE’ in the subsequent section ‘Secondary outcome measures’). Finally, a subjective measure of compliance will be collected from participants from all schools during post-intervention testing using a brief three-item survey asking whether (1) PE lessons start with a warm-up, (2) PE lessons include bursts of VPA that raise their heart rate and make them feel out of breath and (3) participants take part in warm-ups or VPA bursts if asked by PE teachers.

### Blinding

Researchers assessing secondary outcomes are blinded to intervention/control status of schools, but those who are visiting schools to collect fidelity measures and provide top-up intervention training are not blinded. Schools were informed of their status following baseline measurements, so that intervention school teachers could receive training and deliver the intervention.

### Outcome measures

#### Primary outcome measure

The primary outcome measure is academic attainment, assessed by the Progress Test in Maths (PTM), Level 13 [38]. To reduce the test burden, NatCen will administer the test to a random selection of half of all form groups per school, post-intervention only [37].

#### Secondary outcome measures

##### Cardiorespiratory fitness

Fitness is being assessed using the 20-m multistage shuttle run test [39]. This well-validated test [40] measures participants’ maximum aerobic capacity. Schools with a policy of not using the multistage fitness test are completing the Cooper Run Test [41], which requires pupils to run or walk as far as possible in 12 min around a measured track (between 80 and 400 m). Fitness measures are being collected once at baseline and once during the summer term. The tests are conducted by PE teachers during regular PE lessons to minimise the burden on participants and to avoid disrupting other classes.

##### Cognitive and mental health assessments

Cognitive and mental health measures are being collected once at baseline and once during the summer term at the end of the intervention. All cognitive assessments and health questionnaires have been programmed in JavaScript using jsPsych [42], a JavaScript library for running behavioural experiments in a web browser that allows for accurate response time measurements [43]. Only participants can access the assessments, using a personal ID number. Once the assessments are completed, summary measures are created and temporarily stored against participants’ personal IDs in a secure database before being transferred to a second, secure research database, against private ID numbers to ensure anonymisation.

##### (a) Cognitive measures

Cognitive performance is being assessed with tests of processing speed, visual relational memory and core executive functions (working memory, inhibition and cognitive flexibility) [44] that are well validated in children and have been shown to be sensitive to the effects of PA [11, 45]. The cognitive battery takes approximately 50 min to complete and comprises the following tasks:

1. Processing speed is assessed using a simple reaction time task.
2. Visual relational memory performance is assessed with a modified version of a previously described paradigm [10].
3. Inhibitory control is assessed with a modified version of the flanker task [46].
4. Working memory is assessed using a modified version of the visual 2-back task [47, 48].
5. Cognitive flexibility is assessed using a modified version of the colour-shape switch task [46].

Details of each task are provided in Additional file 2.

The task order is pseudo-randomised across participants within schools: all participants start with the reaction time task (i.e. processing speed), followed by the remaining four tasks in a random order (see Additional file 2 for details on the randomisation process). Teachers were instructed to ask pupils to complete the tasks at home, as pilot tests in three schools indicated that the local IT environment (e.g. bandwidth) may not be able to cope with large groups of pupils (> 20) completing the tasks at the same time, and it was deemed a logistical burden on schools to set the tasks during school lessons. Nevertheless, some schools have decided to complete the tasks during school time, as they felt this was easier to manage.

##### (b) Mental and physical health measures

The mental and physical health questionnaire takes approximately 20 min to complete and is intentionally kept brief so that it can be easily completed in school. The questionnaire comprises the following core measures:

1. *Mental health* is assessed with the Strengths and Difficulties Questionnaire (SDQ) [49].
2. *Global self-esteem* is measured using the global self-esteem scale of the short version of the Physical Self-Description Questionnaire (P-SDQ) [50].
3. *Physical self-esteem* is measured using the physical self-esteem scale of the short version of the P-SDQ [50].
4. *Global health* is measured using a single item on a 5-point scale drawn from the Health Behaviour in School-Aged Children (HBSC) survey [24]: ‘In general, how would you say your health is?’ (5 = excellent to 1 = poor).
5. *Physical activity* is measured with a single item [51]: ’In the past week, on how many days have you done a total of 60 minutes or more of physical activity, which was enough to raise your breathing rate? This may include sport, exercise, and brisk walking or cycling for fun, or to get to and from places’.
6. *Habitual physical activity over the past 6 months* is measured with a single item drawn from previous studies [52]: ’Thinking about the past six months, how often have you been physically active for an hour every day during a typical week in school term?’ (1 = never to 7 = always).

We additionally explore the effect of the intervention on daytime sleepiness, PE enjoyment and psychological variables linked to daily MVPA.

7. *Daytime sleepiness.* During adolescence the circadian system delays and lengthens, causing sleep timing to shift later [53], which can lead to daytime sleepiness [54] and behavioural issues including school lateness, absenteeism and, ultimately, poor academic performance [55–57]. Based on evidence of a positive relationship between VPA and sleep quality and quantity in young adults [58] and suggestions that exercise programmes might improve aspects of adolescent sleep [59, 60], the study will investigate the intervention’s impact on daytime sleepiness using the Paediatric Daytime Sleepiness Scale (PDSS) [61].
8. *PE enjoyment.* To investigate the acceptability of the intervention with Year 8 pupils, we measure PE enjoyment with a single item [62]: ’I enjoy PE.’ (1 = strongly disagree to 7 = strongly agree).
9. *Psychological variables linked with daily MVPA.* Understanding the extent to which a programme of VPA during PE might impact psychological variables with potential to explain variance in overall daily MVPA is important, given the low adolescent activity levels [24]. We use the Theory of Planned Behaviour [63, 64] and the Prototype Willingness Model [65] to investigate whether the intervention changes attitudes and beliefs about daily MVPA and active types of people. All items relating to constructs from the Theory of Planned Behaviour and the Prototype Willingness Model were developed according to established theoretical [63, 64] and practical [66, 67] guidelines. An overview of the measures is provided in Additional file 2.

##### Physical activity during PE

Objective measures of VPA during PE are being collected in each school once during regular lessons at baseline (May–July 2017, and at some remaining schools in September 2017) and again during lessons in spring term (March–May 2018). Each school is visited at each timepoint to test at least 50% of the pupils in the year group. Measures are taken using the Axivity AX3, a wrist-worn tri-axial accelerometer designed by Open Lab, Newcastle University, UK. Trained research assistants show pupils how to fit the AX3 on their non-dominant wrist. The devices are attached after pupils change for PE and collected before they change back into school clothes. Class average minutes of MPA and VPA during active lesson time are calculated using established cut-points [68] and standardised, for comparison purposes, to minutes per hour.

##### Covariates

Information about pupils’ ethnicity and language proficiency is collected as part of the online assessments.

##### Brain imaging sub-study

A sub-sample of participants is being recruited to participate in additional testing to investigate the effect of the physical activity intervention on brain structure and function.

The brain imaging sub-study runs in parallel with the main trial, making optimal use of the intervention. Assessments will take place pre-intervention (baseline), immediately post-intervention (t1) and 12 months post-intervention (t2). The brain imaging sub-study consists of two sub-samples, recruited at different times during the trial:

1. The first cohort of 60 participants completed brain-study assessments pre-intervention, and will complete identical assessments post-intervention to investigate pre-to-post intervention changes. Participants will also be invited to complete a set of assessments 1 year following the intervention.
2. A second cohort of 50 participants will be recruited for post-intervention assessments that are similar, but not identical, to the assessments of the first cohort, as well as 1-year follow-up assessments. The addition of this cohort will enable cross-sectional analysis of between-group differences in a second sample.

**Fig. 3:**
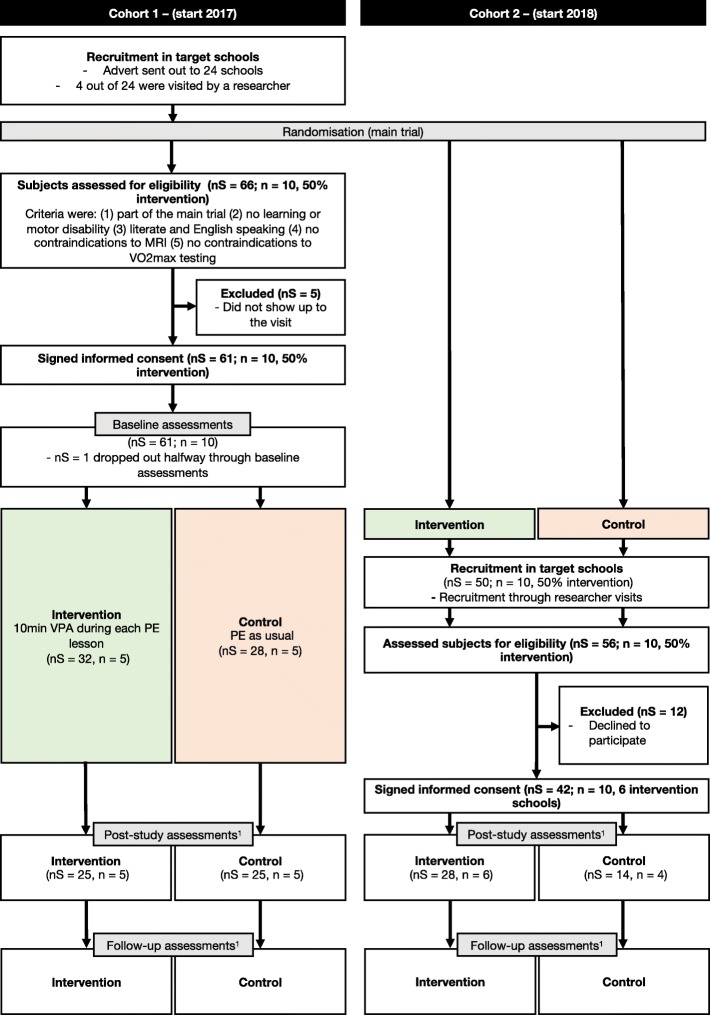
Flow diagram brain imaging sub-study. ^1^Note: post-intervention assessments differ between cohorts 1 and 2, but not within a cohort; see text and Additional file 2: Figure S7. Abbreviations: *MRI* magnetic resonance imaging, *n* number of schools, *nS* number of subjects, *PE* physical education, *VO*_*2*_*max* maximal oxygen consumption, *VPA* vigorous physical activity

Figure 3 shows a diagram of the participant flow, for each cohort, through the study. Details of recruitment for the brain imaging sub-study as well as an overview of assessments per cohort are provided in Additional file 2.

The sub-study has been granted separate ethical approval by the University of Oxford Medical Sciences Inter-Divisional Research Ethics Committee (Registration No. R51313) and was retrospectively registered (19 July 2018) at ClinicalTrials.gov (ID NCT03593863).

##### (a) Brain imaging sub-study outcome measures

1. Magnetic resonance imaging (MRI)

Scanning is carried out at the Wellcome Centre for Integrative Neuroimaging, Oxford, using a 3T Siemens Magnetom Prisma (Erlangen, Germany) scanner with a 32-channel head coil. The MRI protocol comprises both functional and structural sequences, takes approximately 55 min to complete and includes (1) T1-weighted (T1w) structural MRI, (2) resting-state functional MRI (rs-fMRI), (3) diffusion-weighted MRI (DW-MRI), (4) quantitative fast low angle shot (FLASH) MRI, (5) pseudo-continuous arterial spin labelling (pCASL) and (6) MR angiography (MRA; at post-intervention only). Full details of the MRI protocol are provided in Additional file 2.

2. Cognitive measures

Cognitive performance is assessed using tests of visual short-term memory (object-location task [69–71]), relational memory (relational memory task [10]), cognitive flexibility (colour-shape switch task [46]) and planning (Tower of London task [72–74]). Details of the cognitive assessments are provided in Additional file 2. The tests are completed in the lab at baseline (cohort 1), post-intervention (cohorts 1 and 2) and follow-up (cohorts 1 and 2). Cohort 1 completes tests of visual short-term memory and mental associations as well as relational memory and cognitive flexibility. The latter tasks are taken from the main trial and repeated in the brain imaging sub-study to (a) obtain a well-controlled, lab-based measure of the task for cross-validation, and (b) ensure that all brain-study participants will have a score on this task, independent from their main trial completion. Cohort 2 completes tests of visual short-term memory and planning.

3. Mental and physical health measures

A comprehensive set of online questionnaires is completed by all participants at baseline (cohort 1), post-intervention (cohorts 1 and 2) and follow-up (cohorts 1 and 2).

- *Attention-deficit hyperactivity disorder (ADHD)* symptoms are assessed by the ADHD rating scale IV [75], completed by a parent of the participant.
- *Puberty status* is assessed using the Pubertal Development Rating Scale [76], a self-report measure of physical development for youths under the age of 16.
- *Mood* is assessed using the abbreviated Profile of Mood States (POMS) [77]. The abbreviated POMS scale is a well-validated questionnaire that contains 40 self-report items on a 5-point Likert scale.
- *Sleep* is assessed using the Cleveland Adolescent Sleepiness Questionnaire (CASQ [78] and the Sleep Condition Indicator (SCI) [79]. The CASQ is a self-report scale to measure excessive daytime sleepiness. The SCI is an 8-item self-report measure of insomnia symptoms, based on Diagnostic and Statistical Manual of Mental Disorders, 5^th^ edition (DSM-5) criteria, and it captures both sleep problems and daytime functions.
- *Mental health* is assessed with the SDQ [49].
- *General health and behaviour* are assessed using various items of the HBSC survey (2009/2010) [80], capturing body image, eating behaviour, oral health, physical activity, leisure time activity, substance use, family structure, peer relationships, self-rated health and socio-economic environment.

4. Psychological variables linked with daily MVPA.

The study also includes exploratory explicit and implicit measures of psychological variables linked to daily average PA, using the frameworks set out in the Theory of Planned Behaviour [63, 64] and the Prototype Willingness Model [65]. Additional details are provided in Additional file 2.

5. Physical activity, fitness and gait
  - Physical activity

Daily average MVPA during a typical week in school term is measured using the Axivity AX3 accelerometer. A 7-day protocol (5 weekdays and 2 weekend days) is sufficient to obtain a reliability coefficient of 0.8 or above when assessing adolescent PA [81].

(b) Cardiorespiratory fitness

Cardiorespiratory fitness is measured using a standard maximal oxygen consumption (VO_2_max) incremental step test, in which the rate of oxygen consumption is measured while the participant cycles on an ergometer. Resistance is increased every minute until the participant reaches volitional exhaustion or is unable to maintain a cadence of 60 revolutions per minute (rpm). Additional details are provided in Additional file 2.

(c) Gait

Participants walk over a 10-m obstacle-free and flat surface walkway, while wearing a single inertial measurement unit (IMU, LPMS-B, Life Performance Research, Tokyo, Japan), to obtain temporal and spatial gait parameters [82, 83]. Participants perform two consecutive 10-m walks, taking approximately 20 s to complete.

### Sample size/power calculation

The primary intention-to-treat analysis will compare maths test performance between intervention and control schools, for which NatCen has performed a power calculation [36]. The initial power calculation, which demonstrated a range of minimum detectable effect sizes (MDESs) for various achieved samples [36], has been updated (in February–March 2018) to reflect school drop-out (*n* = 18 schools) and a revised set of assumptions [37]. The newly computed MDES is equal to 0.24 standard deviations. The MDES would have been 0.21 standard deviations if all 106 recruited schools had participated in the trial. Power calculations were performed in PowerUp! [84].

### Data analysis plan

In accordance with the Consolidated Standards of Reporting Trials (CONSORT) 2010 statement for cluster-randomised controlled trials guidelines [85], the cluster and participant flow will be reported. Descriptive statistics of recruitment and drop-out will be provided on both the cluster and subject levels. Further, completeness of, and adherence to, the intervention will be reported on a cluster-level only. Baseline characteristics of the randomised groups will be summarised across schools and/or pupils, and will be presented on a pupil level where appropriate.

The primary intention-to-treat analysis comparing maths test performance between intervention and control schools will be conducted by NatCen and is described elsewhere [37]. To account for differences between pupils at baseline, baseline maths performance (Key Stage 2 test score) will be collected from the National Pupil Database (NPD). Analysis of secondary outcome measures will be conducted on an intention-to-treat basis. Per-protocol analyses of secondary measures will also be reported.

Multilevel modelling will be used, as these analyses take the hierarchical structure of the data into account (i.e. pupils within schools) [86]. The models will be used to assess the effect of the intervention, as well as to explore associations between secondary outcome measures, and will adjust for various confounding factors (e.g. sex, socio-economic status, school gender status). Structural equation models (SEMs) may be used for mediation analyses.

MRI data collected as part of the brain imaging sub-study will be analysed and processed using tools from MrTrix3, MATLAB, the FMRIB Software Library (FSL), statistical parametric mapping (SPM) and FreeSurfer, employing parametric and non-parametric statistical analysis methods where appropriate. Details of data (pre) processing and analysis are provided in Additional file 2.

## Discussion

The primary aim of the Fit to Study trial is to investigate the impact of a one academic year (10 months) VPA programme delivered during PE on academic performance in Year 8 pupils. This protocol describes the secondary aims of the trial, including the effect of the intervention on cardiorespiratory fitness, cognitive performance, mental health and the brain. The findings of this trial will contribute to the growing evidence base of the effects of PA on academic achievement [9, 19, 87, 88] and will advance our understanding of the effect of PA on a range of variables — fitness, cognition, mental health and the brain — that are thought to moderate or mediate this relationship. Although many of these variables have individually been linked to PA [4, 11, 60, 89], their role in PA-academic achievement relationships is still unclear.

The early years of secondary school are a period of dynamic neurobiological change. Evidence that additional VPA during school PE has a positive impact on the brain, cognitive function and attainment has the potential to help policymakers and educators steer the developmental course in a positive direction. The Fit to Study intervention could offer a feasible method of increasing VPA without disrupting the curriculum. Further potential advantages are its scalability and ease of dissemination, which are important if the intervention is to be used more widely in the future [90]. The intervention may be incorporated in PE lessons without taking up additional PE time; it should not interfere with the curriculum and does not require extensive training of teachers.

The results could provide further evidence that school is an effective setting for increasing adolescent PA and positively influencing their PA behaviour [91] irrespective of their parents’ PA behaviour or socio-economic status. This is important, given that pupils from deprived backgrounds often have lower PA levels [92–94] and are more difficult to reach via non-school interventions. The findings could also add to evidence on whether PA interventions during PE, including those that supplement PE lessons with vigorous PA, are effective at increasing PA levels [95, 96] and improving cognition [11].

The findings of the Fit to Study trial will provide valuable information for other research groups and organisations looking to design and implement school-based PA interventions. In addition, the trial has the potential to inform the political and scientific debate regarding the role of schools in promoting PA among adolescents.

### Dissemination

The findings of this trial will be published in peer-reviewed journals, irrespective of the direction or magnitude of the results, and will also be presented at national and international scientific meetings. If permitted by journal policies, the results will be made available online wherever possible.

### Trial status

Recruitment of schools for the main trial is complete, the intervention is being delivered and data collection for secondary measures is underway. Recruitment of participants for the brain study is underway.

The trial sponsor is the University of Oxford. The Protocol version is 1.

## Additional files

Additional file 1: Completed SPIRIT (2013) checklist. (DOC 137 kb)

Additional file 2: Supplementary information, providing additional information about the main trial outcome measures, as well as brain imaging sub-study recruitment, outcome measures and data analysis. (DOCX 3944 kb)

## Abbreviations

ADHD: Attention-deficit hyperactivity disorder
CASQ: Cleveland Adolescent Sleepiness Questionnaire
CONSORT: Consolidated Standards of Reporting Trials
DW-MRI: Diffusion-weighted magnetic resonance imaging
FLASH: Fast low angle shot
FSL: FMRIB Software Library
FSM: Free school meals
HBSC: Health Behaviour in School-aged Children
HR: Heart rate
MDES: Minimum detectable effect size
MRA: Magnetic resonance Angiography
MRI: Magnetic resonance imaging
MVPA: Moderate-to-vigorous physical activity
NFER: National Foundation for Educational Research
NPD: National Pupil Database
PA: Physical activity
pCASL: Pseudo-continuous arterial spin labelling
PDSS: Paediatric Daytime Sleepiness Scale
PE: Physical education
POMS: Profile of Mood States
P-SDQ: Physical Self-Description Questionnaire
PTM: Progress Test in Maths
RCT: Randomised controlled trial
rpm: Revolutions per minute
rs-fMRI: Resting-state functional magnetic resonance imaging
SCI: Sleep Condition Indicator
SDQ: Strengths and Difficulties Questionnaire
SPIRIT: Standard Protocol Items: Recommendations for Interventional Trials
SPM: Statistical parametric mapping
T1w: T1-weighted
VO_2_max: Maximal oxygen consumption
VPA: Vigorous physical activity
