# Supplementary material for: Effects of a programme of vigorous physical activity during secondary school physical education on academic performance, fitness, cognition, mental health and the brain of adolescents (Fit to Study): study protocol for a cluster-randomised trial
Source: Trials. 2019 Apr 2;20:189. doi: 10.1186/s13063-019-3279-6 (PMC6444886; doi:10.1186/s13063-019-3279-6)
Supplement: Supplementary file 2 — Supplementary information, providing additional information about the main trial outcome measures, as well as brain imaging sub-study recruitment, outcome measures and data analysis. (DOCX 3944 kb) [file 13063_2019_3279_MOESM2_ESM.docx]

Supplementary information for

**Effects of a programme of vigorous physical activity during secondary school physical education on academic performance, fitness, cognition, mental health and the brain of adolescents (Fit to Study): study protocol for a cluster-randomised trial**

T.M. Wassenaar^1^, C.M. Wheatley^1^, N. Beale^2^, P. Salvan^1^, A. Meaney^2^, J.B. Possee^2^, K. E. Atherton^1^, J.L. Duda^3^, H. Dawes^2^, H. Johansen-Berg^1^

1. Wellcome Centre for Integrative Neuroscience, FMRIB Centre, Nuffield Department of Clinical Neurosciences, University of Oxford, John Radcliffe Hospital, Oxford OX3 9DU, UK
2. Centre for Movement, Occupational and Rehabilitation Sciences (MOReS), Oxford Brookes University, Headington Campus, Oxford OX3 0BP, UK
3. School of Sport, Exercise and Rehabilitation Sciences, University of Birmingham, Birmingham B15 2TT, UK

Correspondence to be addressed to: [heidi.johansen-berg@ndcn.ox.ac.uk](mailto:heidi.johansen-berg@ndcn.ox.ac.uk)

1. **Main trial**
   1. **Randomisation of cognitive assessments**

The order of cognitive assessments was pseudo-randomised by the database developer who is not involved in the analysis of the data. Randomisation was performed per school to ensure a balance in possible task sequences (a total of 24, given that participants start with the reaction time task) across participants, both within and between schools. For each school, participants were sorted randomly and then assigned a number between 1 and 24 (in order), corresponding to each of the possible task sequences, until every participant was allocated a task sequence. Thi process was performed separately for baseline and post-intervention assessments in Microsoft Excel.

- 1. **Outcome measures**
     1. Cognitive assessments

1. Simple reaction time task

Processing speed is being assessed with a simple reaction time task (Figure S1). The task comprises fixation trials and a maximum of 40 target trials. During fixation trials, a black fixation cross is presented centrally for either 1000ms, 2000ms, 3000ms, or 4000ms. The stimulus duration is determined at random, with equal occurrence of each duration. If the participant responds while the cross is on the screen, they receive feedback telling them to wait until they see the target stimulus before pressing, followed by another fixation trial. If the participant does not respond while the cross is on the screen, a target trial is run. During the target trial, a red triangle is presented centrally on a white screen. The triangle disappears once the participant responds by pressing the spacebar on the keyboard. Responses between 100-3000ms are considered valid. The task terminates after 240000ms, irrespective of whether all 40 target trials have been completed. Participants complete a practice block of four target trials and as many fixation trials as needed.

**Figure S1.** Illustration of the simple reaction time task.

1. Flanker task

A modified version of the Flanker task is being used to assess inhibitory control [1], an aspect of executive function. In the task, participants respond as quickly and accurately as possible to the direction of a centrally-presented target arrow which is flanked by an array of arrows that are either congruent (>>>>>) or incongruent (<<><<) to the central target stimulus (Figure S2). Participants are instructed to press the ‘F’ key if the target stimulus points to the left, and the ‘J’ key if the stimulus points to the right. The task consists of two blocks of 52 trials each. Each block contains four trial types: congruent left, congruent right, and incongruent left and incongruent right. Within a block, a total of 13 trials of each type is presented in random order. A trial starts with a blank screen for 500ms, followed by the stimulus array for 333ms and another blank screen for 1067ms. Responses within 200 and 1400ms after stimulus onset are considered valid. At the beginning of the task, participants are given three practice blocks of eight trials each: (1) target stimuli only, with feedback, (2) target and flanked stimuli, with feedback, and (3) target and flanked stimuli without feedback.

**Figure S2.** Illustration of the modified Erickson Flanker task­­­.

1. Visual 2-back task

A modified version of the visual 2-back task [2,3] is being used to assess working memory. Stimuli are pseudo-random sequences of celestial symbols (i.e. sun, moon, rainbow, snowflake and star) presented centrally on a white screen (Figure S3). Participants are instructed to monitor each stimulus and to identify as quickly and accurately as possible whether the stimulus is identical to the one that appeared two trials ago. When the current stimulus matches the previously observed stimulus, the participant must press the ‘F’ key with their left index finger to indicate a positive response; in case of a mismatch, the participants should press the ‘J’ key. The task consists of two blocks of 26 trials each. Of the last 24 trials in each block, eight (one third) are target trials and 16 are foil trials. Whether the current trial is a target or foil is randomly decided at the start of each trial. For target trials, the stimulus from two trials ago is presented on the screen. For foil trials, one of the other stimuli, with the exception of stimuli that were presented one or two trials ago, is selected at random. A trial starts with a blank screen for 500ms, followed by the stimulus for 1500ms and another blank screen for 1000ms. Responses within 150 and 2500ms after stimulus onset are considered valid. At the beginning of the task, participants are given a demonstration of the task to help their understanding. To test their understanding, participants are asked to complete a hypothetical sequence of stimuli by indicating whether the current stimulus would be a target or a foil. If the participant passes the understanding test (<2 errors), they complete a practice block of 12 trials. Practice includes feedback about their accuracy and response time, e.g. a prompt is shown for too slow responses.

**Figure S3.** Illustration of the visual 2-back task.

1. Colour-shape switch task

To assess cognitive flexibility, a modified version of the colour-shape switch task is being used [1]. In the first part of the task, participants are required to learn a set of response actions related to particular attributes of a centrally presented character: (1) its shape (circle or square) and (2) its colour (blue or green). Then, participants are instructed to use a rule-based cue – the direction of the character’s arms – that governs what attribute to pay attention to, requiring them to flexibly shift between attributes and corresponding response actions. In the task, participants complete two blocks of 32 homogeneous trials each; one block of colour only, and one block of shape only (Figure S4). The order of these blocks is randomised. Participants are instructed to pay attention to the centrally presented character and respond as quickly and as accurately as possible by pressing the ‘F’ key on the keyboard with their left index finger if the character is blue or a circle or the ‘J’ key with their right index finger if the character is green or a square. During the heterogeneous condition, participants have to flexibly shift their attention towards the correct attribute (colour or shape) based on the direction of the character’s arms. If the character’s arms are up, the participant has to make a shape-based response; if the arms are down, a colour-based response is required. Following a practice block of 32 trials (16 trials per attribute), participants complete two heterogeneous blocks of 64 trials each. Within each heterogeneous block, the direction of the character’s arms switches every two trials. The stimulus presented on a trial is chosen randomly from the set of stimuli appropriate for that trial type. The different stimuli are presented equally often throughout the task. The first trial of each heterogeneous block is not considered to be a switch or non-switch trial. Within a trial, the stimulus is presented centrally on a black background for 10000ms or until a response is given. Trials are preceded by a 50ms black screen and followed by a red cross for 50ms in the case of an erroneous response. Only responses within 200-6000ms are considered as valid.

**Figure S4.** Illustration of the modified colour-shape switch task, adapted from [1]. Participants were instructed to complete two homogeneous blocks of 32 trials each: (A) shape only, and (B) colour only. (C) Participants then completed two heterogenous blocks of 64 trials each.

1. Relational memory task

Relational memory performance is being assessed with a modified version of a previously described paradigm [4]. The task consists of an encoding phase and recognition phase. During the encoding phase, subjects are presented with two blocks of 18 study trials (i.e. events) containing scene, face and object stimuli (Figure S5). Within a trial, each type of stimulus is shown individually and sequentially for 1300ms in the centre of a white screen. Trials are separated by a centrally presented black fixation cross for 1000ms. Participants are instructed to memorise the stimuli as belonging to a single event. During the recognition phase, memory is tested for associations between stimuli within a trial. A single block of 36 test trials is presented. Half of the test trials are intact, i.e. “target test trials”: the place, face and object all appeared in the same study trial, and the other half of the test trials are rearranged, i.e. “foil test trials”: one of the items is from a different study trial. Stimuli are categorised and split evenly between target and foil test trials to prevent any systematic differences. During a test trial, a place, object and face stimulus are presented simultaneously on a white screen (place on the left, face in the centre and object on the right). Participants are asked to indicate whether all items are from the same event, or whether one of the items does not belong to the event, by pressing a button on the keyboard. Only responses within 200-15000ms are considered as valid. A test trial was followed by a blank screen for 250ms. Prior to the task, participants complete a short practice encoding phase and recognition phase. In the task, the trials are kept the same for all participants, but the order of the study and test trials is randomised. Moreover, there are two different versions of the task, each with its own set of stimuli. Within a school, half of students are randomly assigned version 1, the other half version 2. Participants who completed version 1 at baseline, will complete version 2 post-intervention (and vice versa).

**Figure S5.** Illustration of the modified relational memory task. Participants completed an encoding phase (A), consisting of two blocks of 18 trials each. During the recognition phase (B), participants are asked to indicate whether all items belong to the same event (‘target’) or whether one of the items does not belong to the event (‘foil’).

- - 1. Psychological variables linked to daily MVPA

Government health guidelines suggest that young people accumulate 60 minutes of MVPA per day [5]. To investigate attitudes and beliefs about this volume of activity, we use a description of MVPA drawn from previous research [6,7]. Participants are instructed to think of PA as “*any activity that raises your heart rate and makes you breathe harder and feel warmer. Fast cycling to and from school, running around during school breaks, doing PE and taking part in after-school sports clubs are all examples of physical activity”.* Participants are instructed that *“An hour of physical activity can either be in one block, such as football or netball practice, or several shorter activities that add up to an hour, such as cycling to and from school and running during break and at lunchtime.”*

1. *Attitudes* are measured using a semantic differential scale, anchored with three items drawn from previous research [8,9]: “*For me, being physically active for an hour every day in a typical week during term would be* *stressful/relaxing*; *boring/fun*; *dissatisfying/satisfying*.”
2. *Subjective norms.* Two items (1 = ‘strongly disagree’ to 7 = ‘strongly agree’) assess injunctive norms, or perceived approval of PA, among *people in my family* and *most of my friends,* e.g.: *“People in my family think I should be physically active for an hour every day in a typical week during term.”* A further two items capture descriptive norms, or prevalence of the behaviour among *people in my family* and *most of my friends,* e.g.: *“Most of my friends will be physically active for an hour every day in a typical week during term.”*
3. *Perceived behavioural control* is measured with two items (1 = ‘strongly disagree’ to 7 = ‘strongly agree’). The first taps self-efficacy: *“If I wanted to, I am confident that I could be physically active for an hour every day in a typical week during term.”* The second captures controllability: “*Whether or not I am physically active for an hour every day in a typical week during term is completely up to me.”*
4. *Intention* is measured with a single item (1 = ‘definitely no’ to 7 = ‘definitely yes’): “*I intend to be physically active for an hour every day in a typical week during term*.”
5. *Past behaviour.* Habitual physical activity over the past six months is measured using a single item (1 = Never to 7 = Always): *“Thinking about the past six months, how often have you been physically active for an hour every day during a typical week in term?”* [10].
6. *Prototype favourability****.*** In line with theoretical guidelines [11] participants read a definition of a prototype. Then they are asked to consider active prototypes: “*Think of someone your age who is physically active for an hour every day in a typical week during school term”* and indicate “*how far the following words describe*” this type of person (1 = ‘not at all’ to 7 = ‘extremely’) using four adjectives (*confident, popular, determined, attractive*) drawn from previous research [8].
7. *Prototype similarity.* In line with previous research [12], perceived *similarity* to active and inactive prototypes is measured with single items (1 = ‘very dissimilar’ to 7 = ‘very similar’): “*In general, how similar are you to the type of person who is physically active for an hour every day in a typical week during term?”*
8. *Behavioural willingness.* In line with previous research [13,14], willingness is measured by presenting participants with imaginary situations in school where they could choose to be physically active or inactive in the presence of peers. For each scenario, two items (1 reversed) assess whether participants would be willing to *be* or *avoid* being physically active (1 = ‘extremely unlikely’ to 7 = ‘extremely likely’).

Scenario 1: “*It's lunchtime at school and the teachers have organised an obstacle race on the playing field for a challenge. Some students are taking part and others are watching”.*

Scenario 2*: “It’s morning breaktime at school. Some people you know ask you to join in a game that involves running and jumping. People in your class are standing talking nearby.”*

1. **Brain Imaging sub-study**
   1. ***Recruitment***

Main trial participants were considered eligible for brain-study recruitment. Recruitment for the two cohorts of the brain-study is being done separately.

1. For the first cohort, a sample of main trial schools was identified based on the following criteria: (1) within a 75 miles radius from Oxford, (2) lower socio-economic background, as indicated by the percentage of free school meal (FSM)-eligible Year-7 pupils and postcode-derived index of multiple deprivation. The trial manager, who was not blinded to intervention/control status, ensured a balance between intervention and control schools. Selected schools received an advert to send around to all of their pupils, and one of the researchers, blinded to intervention/control status, visited a sub-sample of four schools to collect expressions of interest from parents and pupils. Individual pupils were considered eligible for the study if the pupil: (1) was part of Fit to Study main trial, (2) had no learning or motor disabilities, (3) was literate and fluent English-speaking, (4) had no contraindication to MRI, including but not limited to certain metallic implants, metallic parts to the eye or a history of claustrophobia, (5) had no contra-indication to a VO_2_max test, as assessed by a modified version of the Physical Activity Readiness Questionnaire (PAR-Q) [15], including any known cardiac or respiratory risk [16]. Subjects were enrolled in the study upon completion of an assent form and completion of a consent form by their parent/guardian.
2. For the second cohort, a sample of main trial schools is being identified based on the following criteria: (1) high completion rates of baseline cognitive, questionnaire and fitness data relative to other schools, (2) responsive to messages from the Fit to Study research team, (3) lower socio-economic background, as indicated by the percentage of FSM-eligible Year-7 pupils or postcode-derived index of multiple deprivation, and, for intervention schools, (4) adherence to the intervention.

From those schools that met criteria, a sub-sample of 10 schools will be visited by a researcher blinded to intervention/control status. Where possible, the researcher will seek expressions of interest (EOI) from a sub-sample of 30 low-fit pupils per school selected by the PE teacher. In other instances, EOI will be sought from the entire year group, and selection for the sub-study will be based on baseline fitness data (i.e. lowest scores on the 20m shuttle run- or Cooper Run test). Selected school that are not interested in the brain-imaging sub-study will be replaced by other schools from the Fit to Study sample that are within reasonable travel distance (max 1.5h) of the brain imaging centre. Inclusion criteria for individual pupils are identical to the criteria for the first group, apart from the VO_2_max screening, which is not part of the assessments for the second group. The trial manager will ensure a balance between subjects from intervention and control schools.

- 1. ***Outcome measures***

An overview of the assessments per cohort is presented in Figure S7.

- - 1. Magnetic resonance imaging (MRI)

The MR-protocol comprises both functional and structural sequences, takes approximately 55 minutes, and includes:

1. T1 weighted (T1w) structural MRI: three-dimensional rapid gradient echo sequence (3D MPRAGE): repetition time (TR) = 1900 ms; echo time (TE) = 3.97 ms; flip angle = 8°; field-of-view (FOV) = 192 mm; voxel size: 1× 1 × 1 mm.
2. Resting-state functional MRI (rs-fMRI): multi-band echo-planar imaging (EPI) sequence; TR = 933ms; TE = 33.40 ms; FOV = 192 mm; 72 slices; voxel size: 2 x 2 x 2 mm; multi-band acceleration factor = 6. For each scan, 644 volumes were acquired. Participants are asked to look at a fixation cross, blink normally, try not to fall asleep and not to think about anything in particular. A field map is also acquired to correct for inhomogeneity distortions.
3. Diffusion-weighted MRI (DWI-MRI): multi-shell, multi-band EPI sequence; *b*-values = 0, 1250, 2500 s/mm^2^, with respectively 11, 60, 60 diffusion weighted directions; TR = 2483 ms; TE = 78.20 ms; FOV = 214 mm; voxel size: 1.75 x 1.75 x 1.75 mm; multi-band acceleration factor = 4. In addition, 4 *b* = 0 s/mm^2^ images are acquired with reversed phase encoding, for the purpose of EPI distortion correction.
4. Quantitative FLASH-MRI [17]: two 3D multi-echo FLASH datasets, one predominantly proton-density weighted (PDw, flip angle = 6°), and one predominantly T1w (flip angle = 21°); FOV = 256 mm; voxel size: 1× 1 × 1 mm; TR = 25 ms; first TE = 2.34 ms; eight equally space echoes, echo spacing = 2.3; GRAPPA acceleration factor = 2 in both phase-encoded directions, with 40 reference lines in each direction. Two single-echo, low-resolution (4 mm isotropic) FLASH scans are acquired before each high-resolution scan; identical FOV; TR = 4 ms; TE = 2 ms; one is acquired receiving on the 32-channel receive head coil, the other receiving on the body coil. To correct for the effect of RF inhomogeneities, the local RF field is mapped using a 2D DAM method with a FLASH readout. For the first eight subjects, FOV was 256 mm, for all other scans, an axial orientation with coverage of 256 mm is also acquired, in order to improve coverage.
5. Pseudo-continuous arterial spin labelling (pCASL) with background pre-saturation [18]: six imaging blocks, each with different post-labelling delays: 0.25, 0.5, 0.75, 1, 1.25 and 1.5 s. Arterial blood is magnetically tagged using a labelling duration of 1.4 s. Other imaging parameters are: single shot EPI; TR = 4100 ms; TE = 14 ms; FOV = 220 mm; voxel size: 3.4 × 3.4 × 4.5 mm. For each scan, 96 volumes (control and tag) are acquired.
6. MR angiography (MRA): using a 3D time-of-flight sequence , TR = 21.0ms; TE = 3.58ms; GRAPPA acceleration factor = 3; flip angle = 18°; FoV = 192mm; 4 slabs with 32 slices each; voxel size: 0.6 x 0.6 x 0.6 mm. MR angiography data is collected at post-intervention only.
   - 1. Cognitive measures

Each cohort completes a battery of cognitive assessments (Table S1). Cohort 1 completes the object-location task, implicit association task, relational memory task and colour-shape switch task. Descriptions of the relational memory task and colour-shape switch task are presented as part of the main trial (section 1.1.1. above). Cohort 2 completes the object-location memory task and the Tower of London task.

| Cohort 1 | Cohort 2 |
| --- | --- |
| - Object-location memory task - Implicit association task - Relational memory task - Colour-shape switching task | - Object-location memory task - Tower of London task |

Table S1. Cognitive assessments completed by each cohort of participants

1. Object-location memory task

Visual short term memory is being assessed using a modified version of the previously described “what was where” object-location task [19–21]. Participants complete a single block of 50 trials. In each trial, subjects are shown three fractal objects, randomly located on an interactive touch screen. The locations of the fractals are determined by a Matlab script (MathWorks, Inc). Participants are instructed to remember the identity of objects and their locations. Following a delay of 1 or 8 seconds, two fractals are displayed on the vertical meridian. One of these fractal objects is part of the memory array (i.e. the target), whereas the other is not (i.e. the foil). Subjects are then asked to touch the remembered object and ‘drag’ it to its remembered location. Memory for object identity (accuracy) and object location are computed, as well as the number of ‘swap or misbinding errors’ (i.e. the correct object was selected, but located near the location of one of the other fractals in the memory array). Prior to the start of the task, participants complete a practice block of 7 trials with either 1 or 3 fractal objects being displayed in the centre of the screen.

1. Tower of London task

Planning is assessed using the Tower of London task [22–24]. In the task, a participant is instructed to move three coloured balls arranged on three pegs of differing heights from their initial position into another pre-specified target configuration. Only one ball can be moved at a time from one peg to another with certain additional restrictions: (1) each of the three pegs could only hold three, two or one balls respectively, and (2) a ball cannot be moved when another ball is lying on top of it. A total of 12 puzzles/problems have to be solved, that differ in difficulty by the number of required moves, ranging from two to five moves. A problem is solved correctly when the target configuration is achieved in the set number of moves, with a total of three attempts per problem. A participant receives three points for solving the problem in one attempt, two points if two attempts are needed, one point if three attempts are required and zero points if more than three attempts are needed. The sum of the points for all 12 problems constitutes their total score (maximum 36 points). Further, the time it takes between problem presentation and the first move is recorded (“planning time”), as well as the time between the first move and the completion of the problem (“execution time”). The task is programmed in Inquisit 4.

- - 1. Psychological variables linked to daily MVPA

The study also includes exploratory explicit and implicit measures of psychological variables linked to daily average PA, using the frameworks set out in the Theory of Planned Behaviour [25,26] and the Prototype Willingness Model

a) The explicit measures set out in 1.1.2 a) above are taken from the main trial and repeated in the brain-imaging sub-study to (1) obtain a well-controlled, lab-based, measure of the task for cross-validation, and (2) ensure that all brain-study participants will have a score on this task, independent from their main trial completion.

b) Implicit Association Test (IAT)

Individuals are thought to have cognitive associations and biases that exist outside conscious awareness [27]. Furthermore, implicit cognitions can influence behaviour [28] and can also, potentially, lend additional predictive power over and above explicit measures [27]. The Implicit Association Test (IAT) [29] is a computer-based sorting task that measures biases [28]. This study uses two IATs developed according to theoretical guidelines [27,29–31] to test the predictive power of implicit prototype evaluations as specified by the Prototype Willingness Model [32]. The Prototype Favourability IAT measures adolescents’ implicit attitudes towards physically active and inactive images (Figure S6). The Prototype Similarity IAT, which also draws from the Self-Concept IAT [33] and the Drinker Identity IAT [34] measures the extent to which participants associate active/inactive characteristics with their own identities. Each IAT assesses the relative strengths of associations between a target concept (*active/inactive)* and a target attribute: *positive/negative* in the favourability IAT; and *me/not* me in the similarity IAT. Participants classify stimuli according to whether they are *active/inactive* or *positive/negative* (or *me/not me*) as quickly as possible. The task rests on the assumption that the more strongly participants associate two concepts (e.g. ‘*active*’ and ‘*positive*’ or ‘*active’* and ‘*me*’) the faster and more accurately they perform.

*Target concepts****.*** The words ‘*active*’ and ‘*inactive*’ represented the concept of the physically active prototype and the physically inactive prototype. To ensure participants understood these concepts, and that they did not confound ‘physically active (inactive)’ with ‘physical activity’, the on-screen instructions also described “*the sort of person your age who is physically active (inactive)*.”

*Attribute concepts****.*** The favourability IAT used ‘positive’ and ‘negative’ as evaluative

attributes, while the similarity IAT used ‘me’ and not me’.

*Stimuli.* The number of stimuli were limited to four in each category of target concept

and attribute (i.e. 16 overall), which is sufficient to maintain validity without sacrificing brevity.

a) *Physically active (inactive) stimuli.* In both IATs, the stimuli used to represent physically active (inactive) prototypes were images of stick-people so that the activity level changed while all other individual characteristics were held constant.

*b) Positive (negative) stimuli.* These were words describing social characteristics. Positive attributes – *confident, popular, determined, attractive –* and negative attributes *– annoying, bossy, judgmental, grumpy –* were drawn from previous research with adolescents [8].

c) *Me (not me) stimuli*. In line with previous research [33], these were synonyms for me/not me: *I, my, mine, self* and *they, them, their, others.*

Both IATs are programmed in *iatgen*, a free, validated tool that runs in Qualtrics and allows researchers to combine IATs and explicit self-report measures in a single survey [35]. Iatgen runs a standard seven-block IAT [29], which takes approximately five minutes to complete. Following the recommended procedure [30], *iatgen* removes participants who record <300ms for more than 10% of responses, and deletes individual trials over 10,000ms. Where participants have made an error, the time spent correcting the mistake is included in latency score. A positive IAT- or D score indicates that participants have relatively stronger associations between the congruent target and attribute ( *‘active’* and ‘*positive*’) than between the incongruent target and attribute (‘*active’* and ‘*negative*’).

| **Inactive** |  | **Active** |  | **Inactive** |  | **Active** |
| --- | --- | --- | --- | --- | --- | --- |
|  |  |  |  | **+** |  | **+** |
|  |  |  |  | **Negative** |  | **Positive** |
|  | 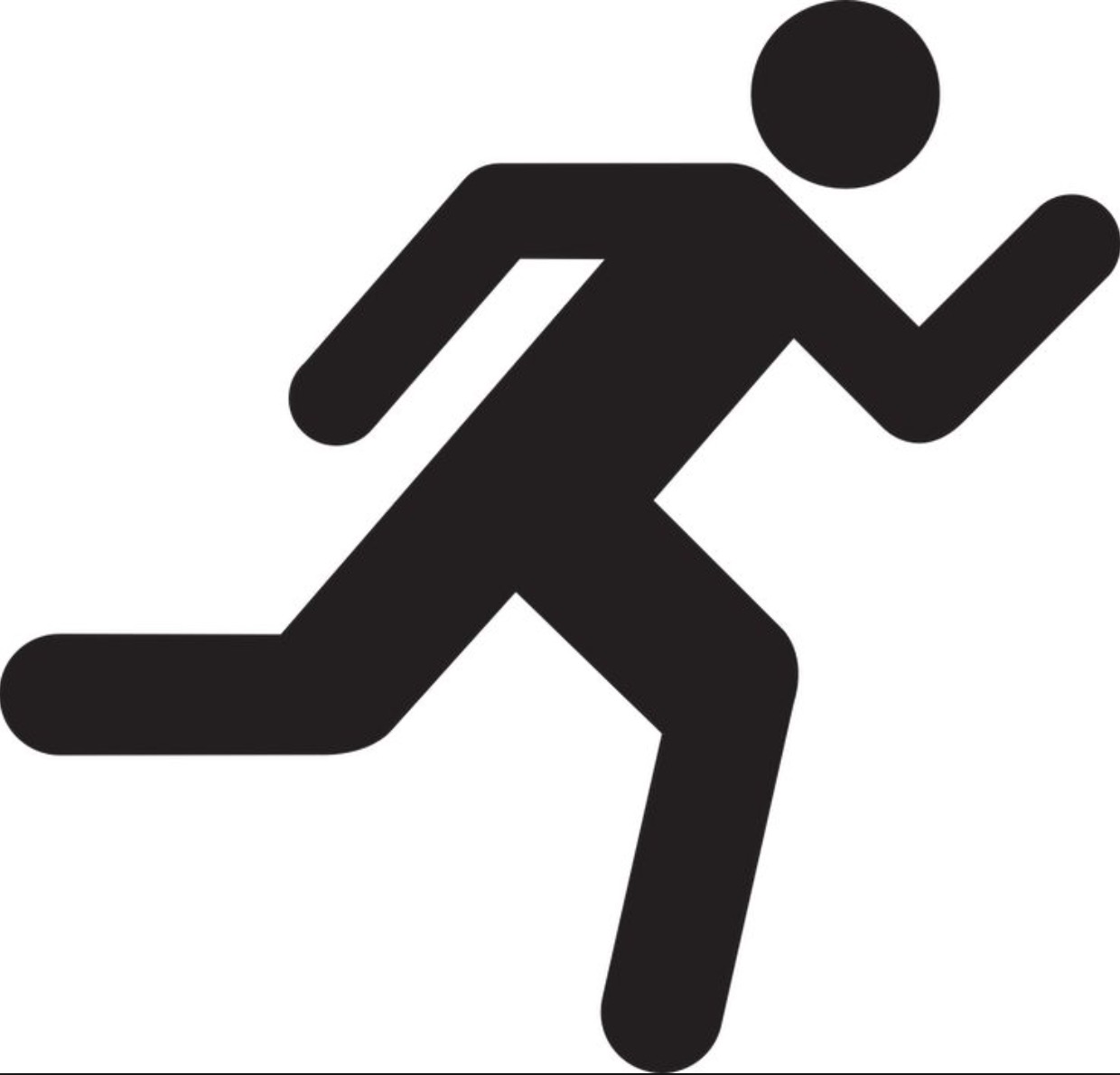 |  |  |  | **Bossy** |  |

**Figure S6.** Illustration of the Prototype Favorability IAT. Participants classify stimuli according to whether they are *active/inactive* or *positive/negative* (or *me/not me*) as quickly as possible.

- - 1. Cardiorespiratory fitness

An incremental step test on a cycle ergometer (Lode Excalibur Sport, Groningen, The Netherlands) is used to measure ventilation, and oxygen and carbon dioxide concentration of the inhaled and exhaled air. Maximal oxygen consumption (VO_2_max), an objective measure of cardiorespiratory fitness, is reached when oxygen consumption remains at a steady state despite an increase in workload. Participants perform the Godfrey protocol [36]**,** which consists of one-minute stages of progressively-higher workload after an initial warm-up phase of two minutes of unloaded cycling. Workload increments are 15W or 20W per minute for, respectively, participants under or over 150cm tall. Participants are instructed to maintain a cadence of 60 revolutions per minute (rpm) throughout. The test is terminated when the subject reaches volitional exhaustion or is unable to maintain a cadence of 60 rpm despite verbal encouragement. The rate of oxygen consumption and heart rate (HR) are measured throughout the test, and rating of perceived exertion is recorded after every increment using the OMNI scale [37]. Only a minority of children exhibit the classic plateau that is used to define VO_2_max in adults [38], therefore our key outcome variable is VO2peak (the average VO2 over the last 30 seconds of the test)[38].We expect that at least 2 of the following 5 criteria will be met [39]: (1) HR > 180 beats/min [40], (2) respiratory exchange ration (RER) > 1.06 [38] (3) RPE at the end of the test > 8 [41], (4) subjective signs of exhaustion [38], and/or (5) whether the ventilatory threshold (VT2), also known as lactate or anaerobic threshold, was reached.

|  |  | **2017** | |  |  |  |  |  | |  | **2018** |  |  |  |  | |  | |  | |  | | **2019** | |  |
| --- | --- | --- | --- | --- | --- | --- | --- | --- | --- | --- | --- | --- | --- | --- | --- | --- | --- | --- | --- | --- | --- | --- | --- | --- | --- |
|  |  | **May** | | **Jun** | **Jul** | **Aug** | **Sep** | **Oct** | |  | **Mar** | **Apr** | **May** | **Jun** | **Jul** | | **Aug** | | **Sep** | |  | | **Jun** | |  |
| **Cohort 1 (n=60)** | Recruitment |  | |  |  |  |  |  | |  |  |  |  |  |  | |  | |  | |  | |  | |  |
|  | Screening & opt-in consent |  | |  |  |  |  |  | |  |  |  |  |  |  | |  | |  | |  | |  | |  |
|  |  |  |  |  |  |  |  |  |  |  |  |  |  |  |  |  |  |  |  |  |  |  |  |  |  |
|  | **Baseline (t0)** |  | |  |  |  |  |  | |  |  |  |  |  |  | |  | |  | |  | |  | |  |
|  | *Cognition Questionnaires VO_2_max & gait MRI* |  | |  |  |  |  |  | |  |  |  |  |  |  | |  | |  | |  | |  | |  |
|  | *7-day actigraphy* |  | |  |  |  |  |  | |  |  |  |  |  |  | |  | |  | |  | |  | |  |
|  | Intervention |  | |  |  |  |  |  | | | | | | | |  | |  | |  | |  | |  | |
|  |  |  |  |  |  |  |  |  |  |  |  |  |  |  |  |  |  |  |  |  |  |  |  |  |  |
|  | **Post-intervention (t1)** |  | | |  |  |  |  |  | |  |  |  |  |  | |  | |  | |  | |  | |  |
|  | *7-day actigraphy* |  |  | |  |  |  |  |  | |  |  |  |  |  | |  | |  | |  | |  | |  |
|  | *Cognition Questionnaires VO_2_max & gait MRI* |  |  | |  |  |  |  |  | |  |  |  |  |  | |  | |  | |  | |  | |  |
|  |  |  |  |  |  |  |  |  |  |  |  |  |  |  |  |  |  |  |  |  |  |  |  |  |  |
|  | **12-month follow-up (t2)** |  | | |  |  |  |  |  | |  |  |  |  |  | |  | |  | |  | |  | |  |
|  | *Cognition* |  | | |  |  |  |  |  | |  |  |  |  |  | |  | |  | |  | |  | |  |
|  | *Questionnaires* |  | | |  |  |  |  |  | |  |  |  |  |  | |  | |  | |  | |  | |  |
|  | *7-day actigraphy* |  | | |  |  |  |  |  | |  |  |  |  |  | |  | |  | |  | |  | |  |
|  |  |  |  |  |  |  |  |  |  |  |  |  |  |  |  |  |  |  |  |  |  |  |  |  |  |
| **Cohort 2 (n=50)** | Recruitment |  |  | |  |  |  |  |  | |  |  |  |  |  | |  | |  | |  | |  | |  |
|  | Screening & opt-in consent |  |  | |  |  |  |  |  | |  |  |  |  |  | |  | |  | |  | |  | |  |
|  |  |  |  |  |  |  |  |  |  |  |  |  |  |  |  |  |  |  |  |  |  |  |  |  |  |
|  | **Post-intervention (t1)** |  |  | |  |  |  |  |  | |  |  |  |  |  | |  | |  | |  | |  | |  |
|  | *7-day actigraphy* |  |  | |  |  |  |  |  | |  |  |  |  |  | |  | |  | |  | |  | |  |
|  | *Cognition Questionnaires Gait  MRI* |  |  | |  |  |  |  |  | |  |  |  |  |  | |  | |  | |  | |  | |  |
|  |  |  |  |  |  |  |  |  |  |  |  |  |  |  |  |  |  |  |  |  |  |  |  |  |  |
|  | **12-month follow-up (t2)** |  | | |  |  |  |  |  | |  |  |  |  |  | |  | |  | |  | |  | |  |
|  | *Cognitive tasks Questionnaires 7-day actigraphy* |  |  | |  |  |  |  |  | |  |  |  |  |  | |  | |  | |  | |  | |  |

**Figure S7**. Schedule of assessments for the brain imaging sub-study, split by cohort

- 1. ***Data analysis plan***

MRI data is preprocessed using tools from FMRIB’s Software Library (FSL) [42], Statistical Parametric Mapping (SPM)[43] and FreeSurfer [44]. Here we present an overview of some of the processing steps per imaging modality.

1. *Structural T1w.* The T1w data is corrected for gradient distortions using gradient distortion correction (GDC) tools developed by Freesurfer and Human Connectome Project teams [45]. The data is then processed using the *fsl_anat* anatomical processing pipeline available within FSL, which includes bias field correction using FAST (FMRIB’s Automated Segmentation Tool), registration to MNI152 standard space T1 template using FLIRT (FMRIB’s Linear Image Registration Tool) [46] followed by FNIRT (FMRIB’s Non-linear Image Registration Tool) [47] and brain-extraction using FNIRT-based registrations. Brain extraction may also be performed using BET (FMRIB’s Brain Extraction Tool) if appropriate [48]. Tissue-type segmentation is then applied using FAST and subcortical structures are modelled using FIRST (FMRIB’s Integrated Registration and Segmentation Tool), and separately using MIST (Multimodal Image Segmentation Tool) [49,50]. Furthermore, subject-specific cortical surface reconstructions and cortical parcellations are derived using FreeSurfer [44].
2. *Resting-state fMRI.* rs-fMRI data is corrected for inter- and intra-volume subject head motion, as well as EPI distortions using FSL’s Topup [51]. Highpass temporal filtering and GDC unwarping are also applied. Registration to each subject’s T1w image is improved by an additional rigid-body registration step aided by a single-band EPI image. Structured artefacts are removed by ICA+FIX processing (independent component analysis and FMRIB’s ICA-based X-noisefier) [52–54]. FSL’s MELODIC (Multivariate Exploratory Linear Optimized Decomposition into Independent Components) tool is used to estimate group-average resting-state networks (RSNs).
3. *Diffusion-weighted MRI.* DWI-MRI data is first corrected for eddy currents, EPI distortions, inter- and intra-volume subject head motion, with outlier-slice replacement, using FSL’s eddy tool [55]. GDP unwarping is applied [56]. Diffusion Tensor Imaging (DTI) fitting is carried out with FSL DTIFIT [57]. Neurite Orientation Dispersion and Density Imaging (NODDI) modelling is estimated using FSL cuDIMOT [58], based on the Bingham-NODDI model [59]. In order to resolve crossing-fibres configurations, multi-shell voxel-wise diffusion is modelled using FSL BedpostX [60]. Probabilistic tractography is carried out with FSL ProbtrackX [57] and major white matter bundles are reconstructed as implemented in FSL AutoPtx [61].
4. *Quantitative MRI* Quantitative MRI data is processed to produce the quantitative maps of myelination and iron level, using the Voxel-Based Quantification (VBQ) toolbox [62] in SPM.
5. *ASL* ASL data is processed using BASIL (Bayesian Inference for Arterial Spin Labelling) [63], a collection of tools available in FSL that creates quantitative cerebral blood flow images from ASL data.
6. *MRA* Angiography data is processed to produce indices of the vessel structure (e.g. vessel radius, density, and tortuosity) . The cerebrovascular system will be segmented using a multi-step segmentation framework [64], followed by calculation of the of the 3D vessel centreline representation and corresponding vessel radius for each voxel of the centreline [65,66].

Further MRI data processing and analysis will be carried out using tools from MrTrix3, MATLAB, FSL, and Freesurfer, employing parametric and non-parametric statistical analysis methods where appropriate.

1. **Abbreviations**

ADHD: Attention-Deficit Hyperactivity Disorder

BASIL: Bayesian Inference for Arterial Spin Labelling

BET: Brain Extraction Tool

CASQ: Cleveland Adolescent Sleepiness Questionnaire

CONSORT: Consolidated Standard for Reporting Trials

DWI – MRI: Diffusion-Weighted Imaging -Magnetic Resonance Imaging

EOI: Expression of Interest

EPI: Echo-Planar Imaging

FAST: FMRIB’s Automated Segmentation Tool

FIRST: FMRIB’s Integrated Registration and Segmentation Tool

FLIRT: FMRIB’s Linear Image Registration Tool

FNIRT: FMRIB’s Non-linear Image Registration Tool

FSL: FMRIB’s Software Library

FSM: Free school meals

GDC: Gradient distortion correction

HBSC: Health behaviour for school aged children

HR: Heart rate

FOV: Field of view

MDES: Minimum detectable effect size

MELODIC: Multivariate Exploratory Linear Optimized Decomposition into Independent Components

MIST: Multimodal Image Segmentation Tool

MRI: Magnetic Resonance Imaging

MVPA: Moderate-to-vigorous physical activity

NFER: National Foundation for Educational Research

NODDI: Neurite Orientation Dispersion and Density Imaging

NPD: National Pupil Database

RER: Respiratory Exchange Ratio

rpm: revolutions per minute

rs-fMRI: resting-state functional Magnetic Resonance Imaging

PA: Physical activity

PAR-Q: Physical Activity Readiness Questionnaire

pCASL: Pseudo-Continuous Arterial Spin Labelling

PDw: Proton Density-weighted

POMS: Profile of Mood States

PTM: Progress Test in Maths

RCT: Randomised Control Trial

SCI: Sleep Condition Indicator

SDQ: Strength and Difficulties Questionnaire

SPM: Statistical Parametric Mapping

T1w: T1-weighted

TR: Repetition Time

TE: Echo Time

MPRAGE: Rapid Gradient Echo sequence

VBQ: Voxel-based quantification

VO_2_max: Maximal oxygen consumption

1. **References**

1. Hillman CH, Pontifex MB, Castelli DM, Khan NA, Raine LB, Scudder MR, et al. Effects of the FITKids Randomized Controlled Trial on Executive Control and Brain Function. Pediatrics. 2014;134:e1063–71.

2. Chen A-G, Yan J, Yin H-C, Pan C-Y, Chang Y-K. Effects of acute aerobic exercise on multiple aspects of executive function in preadolescent children. Psychol Sport Exerc. 2014;15:627–36.

3. Guiney H, Machado L. Benefits of regular aerobic exercise for executive functioning in healthy populations. Psychon Bull Rev. 2013;20:73–86.

4. Chaddock L, Erickson KI, Prakash RS, Kim JS, Voss MW, Vanpatter M, et al. A neuroimaging investigation of the association between aerobic fitness, hippocampal volume, and memory performance in preadolescent children. Brain Res. 2010;1358:172–83.

5. 2018 Physical Activity Guidelines Advisory Committee. 2018 Physical Activity Guidelines Advisory Committee Scientific Report. Washington, DC; 2018.

6. Inchley J, Currie D, Young T, Torsheim T, Augustson L, Mathison F. Health behaviour in school-aged children (HBSC) study: International Report from the 2013/2014 survey. Heal Policy Child Adolesc. 2016;

7. Scott JJ, Morgan PJ, Plotnikoff RC, Lubans DR. Reliability and validity of a single‐item physical activity measure for adolescents. J Paediatr Child Health. 2015;51:787–93.

8. Wheatley CM, Davies EL, Dawes H. Unspoken Playground Rules Discourage Adolescent Physical Activity in School: A Focus Group Study of Constructs in the Prototype Willingness Model. Qual Health Res. 2017;28:624–32.

9. Rivis A, Sheeran P. Social influences and the theory of planned behaviour: Evidence for a direct relationship between prototypes and young people’s exercise behaviour. Psychol Heal. 2003;18:567–83.

10. Hagger MS, Chatzisarantis N, Biddle SJH, Orbell S. Antecedents of children’s physical activity intentions and behaviour: Predictive validity and longitudinal effects. Psychol Heal. 2001;16:391–407.

11. Gibbons FX, Gerrard M, McCoy SB. Prototype perception predicts (lack of) pregnancy prevention. Personal Soc Psychol Bull. 1995;21:85–93.

12. Rivis A, Sheeran P, Armitage CJ. Augmenting the theory of planned behaviour with the prototype/willingness model: Predictive validity of actor versus abstainer prototypes for adolescents’ health‐protective and health‐risk intentions. Br J Heal Psychol. 2006;11:483–500.

13. Davies EL, Martin J, Foxcroft DR. Age differences in alcohol prototype perceptions and willingness to drink in UK adolescents. Psychol Health Med. 2016;21:317–29.

14. Gibbons FX, Gerrard M, Blanton H, Russell DW. Reasoned action and social reaction: willingness and intention as independent predictors of health risk. J Pers Soc Psychol. 1998;74:1164.

15. Canadian Society for Exercise Physiology. Physical Activity Readiness Questionnaire (PAR-Q). 2002.

16. Riebe D, Franklin BA, Thompson PD, Garber CE, Whitfield GP, Magal M, et al. Updating ACSM’s recommendations for exercise preparticipation health screening. Med Sci Sports Exerc. 2015;47:2473–9.

17. Weiskopf N, Suckling J, Williams G, Correia M. MM, Inkster B, Tait R, et al. Quantitative multi-parameter mapping of R1, PD*, MT, and R2* at 3T: A multi-center validation. Front Neurosci. 2013;7:1–11.

18. Okell TW, Chappell MA, Kelly ME, Jezzard P. Cerebral blood flow quantification using vessel-encoded arterial spin labeling. J Cereb Blood Flow Metab. 2013;33:1716–24.

19. Pertzov Y, Dong MY, Peich MC, Husain M. Forgetting What Was Where: The Fragility of Object-Location Binding. PLoS One. 2012;7:e48214.

20. Pertzov Y, Miller TD, Gorgoraptis N, Caine D, Schott JM, Butler C, et al. Binding deficits in memory following medial temporal lobe damage in patients with voltage-gated potassium channel complex antibody-associated limbic encephalitis. Brain. 2013;136:2474–85.

21. Liang Y, Pertzov Y, Nicholas JM, Henley SMD, Crutch S, Woodward F, et al. Visual short-term memory binding deficit in familial Alzheimer’s disease. Cortex. 2016;78:150–64.

22. Shallice T. Specific Impairments of Planning. Philos Trans R Soc B Biol Sci. 1982;298:199–209.

23. van der Niet AG, Smith J, Scherder EJA, Oosterlaan J, Hartman E, Visscher C. Associations between daily physical activity and executive functioning in primary school-aged children. J Sci Med Sport. 2015;18:673–7.

24. Anderson P, Anderson V, Lajoie G. The tower of London test: Validation and standardization for pediatric populatons. Clin Neuropsychol. 1996;10:54–65.

25. Ajzen I. From intentions to actions: A theory of planned behavior. Springer; 1985.

26. Ajzen I. The theory of planned behavior. Organ Behav Hum Decis Process. 1991;50.

27. Greenwald AG, Poehlman TA, Uhlmann EL, Banaji MR. Understanding and using the Implicit Association Test: III. Meta-analysis of predictive validity. J Pers Soc Psychol. 2009;97:17.

28. Strack F, Deutsch R. Reflective and impulsive determinants of social behavior. Personal Soc Psychol Rev. 2004;8:220–47.

29. Greenwald AG, McGhee DE, Schwartz JLK. Measuring individual differences in implicit cognition: the implicit association test. J Pers Soc Psychol. 1998;74:1464.

30. Greenwald AG, Nosek BA, Banaji MR. Understanding and using the implicit association test: I. An improved scoring algorithm. J Pers Soc Psychol. 2003;85:197.

31. Nosek BA, Greenwald AG, Banaji MR. Understanding and using the Implicit Association Test: II. Method variables and construct validity. Personal Soc Psychol Bull. 2005;31:166–80.

32. Gerrard M, Gibbons FX, Houlihan AE, Stock ML, Pomery EA. A dual-process approach to health risk decision making: The prototype willingness model. Dev Rev. 2008;28:29–61.

33. Greenwald AG, Farnham SD. Using the implicit association test to measure self-esteem and self-concept. J Pers Soc Psychol. 2000;79:1022.

34. Gray HM, LaPlante DA, Bannon BL, Ambady N, Shaffer HJ. Development and validation of the alcohol identity implicit associations test (AI-IAT). Addict Behav. 2011;36:919–26.

35. Carpenter TPT, Pogacar R, Pullig C, Isenberg N, Kouril M, LaBouff J, et al. Building and Analyzing Implicit Association Tests for Online Surveys: A Tutorial and Open-source Tool. Manuscr Submitt Publ. 2017;

36. Godfrey S, Mearns M. Pulmonary function and response to exercise in cystic fibrosis. Arch Dis Child. 1971;46:144–51.

37. Utter AC, Robertson RJ, Nieman DC, Kang JIE. Children’s OMNI scale of perceived exertion : walking / running evaluation. Med Sci Sport Exerc. 2002;34:139–44.

38. Winter E, Jones A, Davidson R, Bromley P, Mercer T. Sport and Exercise Physiology Testing Guidelines: Volume II - Exercise and Clinical Testing. The British Association of Sport and Exercise Sciences Guide. London: Routledge; 2007.

39. Cooper CB, Storer TW. Exercise Testing and Interpretation: A Practical Approach. Cambridge: Cambridge University Press; 2001.

40. Schulze-Neick I, Wessel H, Paul M. Heart rate and oxygen uptake response to exercise in children with low peak exercise heart rate. Eur J Pediatr. 1992;151:160–6.

41. Franklin BA, Whaley MH, Howley ET, Balady GJ. ACSM’s Guidelines for Exercise Testing and Prescription (7th ed.). Philadelphia: Lippincott Williams & Wilkins; 2000.

42. Jenkinson M, Beckmann CF, Behrens TEJ, Woolrich MW, Smith SM. Fsl. Neuroimage. 2012;62:782–90.

43. Statistical Parametric Mapping (SPM) [Internet]. [cited 2018 May 1]. Available from: http://www.fil.ion.ucl.ac.uk/spm/

44. Dale AM, Fischl B, Sereno MI. Cortical surface-based analysis: I. Segmentation and surface reconstruction. Neuroimage. 1999;9:179–94.

45. Gradient distortion correction. :Accessed on 14 March 2018. Available from: https://github.com/Washington-University/Pipelines

46. Jenkinson M, Bannister P, Brady M, Smith S. Improved Optimization for the Robust and Accurate Linear Registration and Motion Correction of Brain Images. Neuroimage. 2002;17:825–41.

47. Andersson JLR, Jenkinson M, Smith S. Non-linear registration aka Spatial normalisation FMRIB Technial Report TR07JA2. 2007;22. Available from: www.fmrib.ox.ac.uk/datasets/techrep/tr07ja2/tr07ja2.pdf

48. Smith SM. Fast robust automated brain extraction. Hum Brain Mapp. 2002;17:143–55.

49. Patenaude B, Smith SM, Kennedy DN, Jenkinson M. A Bayesian model of shape and appearance for subcortical brain segmentation. Neuroimage. 2011;56:907–22.

50. Visser E, Keuken MC, Douaud G, Gaura V, Bachoud-Levi AC, Remy P, et al. Automatic segmentation of the striatum and globus pallidus using MIST: Multimodal Image Segmentation Tool. Neuroimage. 2016;125:479–97.

51. Andersson JLR, Skare S, Ashburner J. How to correct susceptibility distortions in spin-echo echo-planar images: application to diffusion tensor imaging. Neuroimage. 2003;20:870–88.

52. Beckmann CF, Smith SM. Probabilistic Independent Component Analysis for Functional Magnetic Resonance Imaging. IEEE Trans Med Imaging. 2004;23:137–52.

53. Salimi-Khorshidi G, Douaud G, Beckmann CF, Glasser MF, Griffanti L, Smith SM. Automatic denoising of functional MRI data: Combining independent component analysis and hierarchical fusion of classifiers. Neuroimage. 2014;90:449–68.

54. Griffanti L, Salimi-Khorshidi G, Beckmann CF, Auerbach EJ, Douaud G, Sexton CE, et al. ICA-based artefact removal and accelerated fMRI acquisition for improved resting state network imaging. Neuroimage. 2014;95:232–47.

55. Andersson JLR, Sotiropoulos SN. Non-parametric representation and prediction of single-and multi-shell diffusion-weighted MRI data using Gaussian processes. Neuroimage. 2015;122:166–76.

56. Miller KL, Alfaro-Almagro F, Bangerter NK, Thomas DL, Yacoub E, Xu J, et al. Multimodal population brain imaging in the UK Biobank prospective epidemiological study. Nat Neurosci. 2016;19:1523–36.

57. Behrens TEJ, Berg HJ, Jbabdi S, Rushworth MFS, Woolrich MW. Probabilistic diffusion tractography with multiple fibre orientations: What can we gain? Neuroimage. 2007;34:144–55.

58. cuDIMOT. :Accessed on 14 Feb 2018. Available from: https://users.fmrib.ox.ac.uk/~moisesf/cudimot/DesignModel.html

59. Tariq M, Schneider T, Alexander DC, Gandini Wheeler-Kingshott CA, Zhang H. Bingham-NODDI: Mapping anisotropic orientation dispersion of neurites using diffusion MRI. Neuroimage. 2016;133:207–23.

60. Jbabdi S, Sotiropoulos SN, Savio AM, Graña M, Behrens TEJ. Model-based analysis of multishell diffusion MR data for tractography: How to get over fitting problems. Magn Reson Med. 2012;68:1846–55.

61. De Groot M, Vernooij MW, Klein S, Ikram MA, Vos FM, Smith SM, et al. Improving alignment in Tract-based spatial statistics: Evaluation and optimization of image registration. Neuroimage. 2013;76:400–11.

62. Callaghan MF, Freund P, Draganski B, Anderson E, Cappelletti M, Chowdhury R, et al. Widespread age-related differences in the human brain microstructure revealed by quantitative magnetic resonance imaging. Neurobiol Aging. 2014;35:1862–72.

63. Chappell MA, Groves AR, Whitcher B, Woolrich MW. Variational Bayesian Inference for a Nonlinear Forward Model. IEEE Trans Signal Process. 2009;57:223–36.

64. Forkert ND, Schmidt-Richberg A, Fiehler J, Illies T, Möller D, Säring D, et al. 3D cerebrovascular segmentation combining fuzzy vessel enhancement and level-sets with anisotropic energy weights. Magn Reson Imaging. 2013;31:262–71.

65. Forkert ND, Fiehler J, Suniaga S, Wersching H, Knecht S, Kemmling A. A statistical cerebroarterial atlas derived from 700 MRA datasets. Methods Inf Med. 2013;52:467–74.

66. Rätsep MT, Paolozza A, Hickman AF, Maser B, Kay VR, Mohammad S, et al. Brain structural and vascular anatomy is altered in offspring of pre-eclamptic pregnancies: A pilot study. Am J Neuroradiol. 2016;37:939–45.
